# Association of Cardiovascular Risk Factors and Coronary Calcium Burden with Epicardial Adipose Tissue Volume Obtained from PET–CT Imaging in Oncological Patients

**DOI:** 10.3390/jcdd11100331

**Published:** 2024-10-17

**Authors:** Carmela Nappi, Andrea Ponsiglione, Carlo Vallone, Roberto Lepre, Luigi Basile, Roberta Green, Valeria Cantoni, Ciro Gabriele Mainolfi, Massimo Imbriaco, Mario Petretta, Alberto Cuocolo

**Affiliations:** 1 Department of Advanced Biomedical Sciences, University Federico II, 80131 Naples, Italy; c.nappi@unina.it (C.N.); andrea.ponsiglione@unina.it (A.P.); carlo.vallone@unina.it (C.V.); roberto.lepre@unina.it (R.L.); luigi.basile@unina.it (L.B.); roberta.green@unina.it (R.G.); valeria.cantoni@unina.it (V.C.); cirogabriele.mainolfi@unina.it (C.G.M.); massimo.imbriaco@unina.it (M.I.); cuocolo@unina.it (A.C.); 2 IRCCS Synlab SDN, 80143 Naples, Italy

**Keywords:** coronary artery disease, coronary artery calcium, epicardial adipose tissue, PET/CT imaging

## Abstract

Whole-body positron emission tomography (PET)–computed tomography (CT) imaging performed for oncological purposes may provide additional parameters such as the coronary artery calcium (CAC) and epicardial adipose tissue (EAT) volume with cost-effective prognostic information in asymptomatic people beyond traditional cardiovascular risk factors. We evaluated the feasibility of measuring the CAC score and EAT volume in cancer patients without known coronary artery disease (CAD) referred to whole-body ^18^F-FDG PET–CT imaging, regardless of the main clinical problem. We also investigated the potential relationships between traditional cardiovascular risk factors and CAC with EAT volume. A total of 109 oncological patients without overt CAD underwent whole-body PET–CT imaging with ^18^F-fluorodeoxyglucose (FDG). Unenhanced CT images were retrospectively viewed for CAC and EAT measurements on a dedicated platform. Overall, the mean EAT volume was 99 ± 49 cm^3^. Patients with a CAC score ≥ 1 were older than those with a CAC = 0 (*p* < 0.001) and the prevalence of hypertension was higher in patients with detectable CAC as compared to those without (*p* < 0.005). The EAT volume was higher in patients with CAC than in those without (*p* < 0.001). For univariable age, body mass index (BMI), hypertension, and CAC were associated with increasing EAT values (all *p* < 0.005). However, the correlation between the CAC score and EAT volume was weak, and in multivariable analysis only age and BMI were independently associated with increased EAT (both *p* < 0.001), suggesting that potential prognostic information on CAC and EAT is not redundant. This study demonstrates the feasibility of a cost-effective assessment of CAC scores and EAT volumes in oncological patients undergoing whole-body ^18^F-FDG PET–CT imaging, enabling staging cancer disease and atherosclerotic burden by a single test already included in the diagnostic work program, with optimization of the radiation dose and without additional costs.

## 1. Introduction

Since 1991, the risk of death from oncological reasons has declined continuously, with an overall drop of 32% when compared to up-to-date rates [1]. On the other hand, while we are assisting such a cancer-related death decline due to the refinement of both diagnostic capabilities and therapeutic armamentarium, cardiovascular diseases currently remain the main cause of death, and it is precisely oncological patients who are at a higher risk of developing coronary artery disease (CAD) compared to the general population. This finding is partially due to common CAD and cancer risk factors and partially related to specific oncological therapeutic approaches such as thoracic external beam radiotherapy and chemotherapy, including anthracycline treatments [2,3]. Hence, in cancer patients, especially in those with a high probability of long-term survival, it is important to assess cardiovascular risk. The role of standard modifiable cardiovascular risk factors in determining CAD has been widely investigated [4,5,6]. On the other hand, imaging data may contribute to improving CAD risk stratification. Coronary artery calcium (CAC) burden, obtained using different methods including unenhanced computed tomography (CT), has emerged as the most predictive single cardiovascular risk marker in asymptomatic persons, capable of adding predictive information beyond traditional cardiovascular risk factors, with cost effectiveness [7,8].

Available data from large population analysis studies are consistent with the concept that CAC testing represents a reasonable option to risk stratify cardiovascular impairment without increased costs [3,9]. On the other hand, a recent meta-analysis evaluated the incremental benefit of adding CAC scoring to standard cardiovascular disease risk calculators, considering six studies with a total of 1043 cardiovascular events among 17,961 participants [10]. Although the results indicated that CAC scoring provided some additional discrimination beyond traditional risk assessment equations, with this improvement being relatively consistent across the studies, the authors highlighted that this slight improvement is often counterbalanced by the associated costs, incidental findings, and radiation exposure, affirming that it is unclear which patients would truly benefit. There is also growing evidence that the quantification of epicardial adipose tissue (EAT) by cardiac imaging may play a significant role in CAD risk stratification in patients with suspected atherosclerosis [11,12,13,14,15].

With regard to the oncological population, Lee et al. [16] suggested that the EAT area on low-dose chest CT could be used to predict coronary atherosclerosis in an asymptomatic population considered for lung cancer screening. The potential role of EAT as a biomarker of cancer-related therapy cardiotoxicity has also been proposed [17]. A recent study evaluated changes in the EAT in patients with follicular lymphoma treated with two therapeutic approaches and with different potential cardiotoxicities and demonstrated that an EAT increase may be a marker for the early detection of myocardial damage [18].

The purpose of the present study was to evaluate the feasibility of measuring, in a cost-effective manner, CAC scores and EAT volumes in patients referred to whole-body ^18^F-FDG PET–CT imaging, regardless of the main clinical question. We also assessed the association of cardiovascular risk factors and coronary calcium content with the EAT volume obtained from whole-body positron emission tomography (PET)–CT imaging in oncological patients without known CAD.

## 2. Materials and Methods

### 2.1. Study Population

From February 2022 to March 2023, 109 consecutive patients were enrolled. Only patients undergoing whole-body PET–CT imaging with ^18^F-fluorodeoxyglucose (FDG), as part of their diagnostic or follow-up program, for oncological reasons were included. The following data were considered for exclusion criteria: previously diagnosed CAD including a history of myocardial infarction (chest pain or equivalent symptom complex, positive cardiac biomarkers, or typical electrocardiographic changes), of percutaneous coronary intervention, or of coronary artery bypass grafting; severe valvular or congenital heart disease; and the presence of implantable cardiac devices.

As part of the baseline examination, clinical information including traditional cardiovascular risk factors, current smoker status, hypercholesterolemia, diabetes mellitus, and hypertension was collected. Current smoking was defined if patients had regularly smoked (≥1 cigarette per day) within the past month before imaging. Hypercholesterolemia was defined as having a previous diagnosis of the mentioned condition, previous or ongoing oral low-density lipoprotein cholesterol (LDL-C) lowering treatment, an LDL-C concentration of 3.5 mmol/L or higher, or a total cholesterol concentration of 5.5 mmol/L or higher at the time of imaging. Diabetes (type 1 and type 2) was identified when patients demonstrated a previous diagnosis of diabetes or current glucose lowering therapy [19]. Hypertension was defined as a blood pressure > 140/90 mm Hg or current anti-hypertensive therapy [20]. A familiar history of premature CAD was noted in the case of a diagnosis of CAD in a first degree relative prior to or at 55 years of age in men or 65 years in women [21]. Patients reporting anginal symptoms were defined as symptomatic. Chest pain was classified as non-anginal chest pain, atypical angina, or typical angina [22]. The review committee of our institution approved this study, and all patients gave informed consent.

### 2.2. PET/CT Imaging

All patients were required to fast for at least 6 h prior to unenhanced PET/CT imaging, and in all subjects included in this study blood glucose levels were <180 mg/dL at the time of the ^18^F-FDG injection. ^18^F-FDG PET/CT unenhanced images were acquired using a PET/CT Ingenuity TF (Philips Healthcare, Best, The Netherlands) 60 min after the tracer administration (activity range 200–300 MBq, according to body weight) [23,24].

All examinations were performed in a three-dimensional mode. An emission scan was completed, from the upper thigh to the base of the skull, in the caudocranial direction (4 min for each bed position). Iterative image reconstruction was finalized with an ordered subset expectation maximization algorithm (2 iterations, 28 subsets). A T 4-slice multi-detector helical CT scanner was used (detector row configuration, 4 × 5 mm; pitch, 1.5; gantry rotation speed, 0.8 s per revolution; table speed, 30 mm per gantry rotation; 140 kV and 80 mA). Using filtered back projection CT reconstructed images (Gaussian filter with 8 mm full width at half maximum) to match the PET resolution, attenuation-corrected emission data were attained. Transaxial, sagittal, and coronal images and co-registered images were evaluated using Philips IntelliSpace Portal, Image and information management software version 9.0 (Philips Medical Systems, Veenpluis, Best, The Netherlands). The co-registered CT images were recovered and estimated with a dedicated workstation for post-processing and analysis as previously illustrated [25].

The CT studies were analyzed by consensus from experienced nuclear medicine physicians and radiologists blinded to the PET results. Calcium was defined as the presence of at least 3 contiguous pixels with a density > 130 HU. The total calcium load in the coronary arteries was measured based on the scoring algorithm proposed by Agatston et al. [26]. CAC scores were estimated separately for left anterior descending, left circumflex, and right coronary arteries, and they were then summed to obtain a total CAC score. For quantification of the EAT volume, the image processing started at the level of the pulmonary trunk and ended at the level of the inferior diaphragmatic surface of the heart to manually trace pericardial borders. The area outside the traced pericardium was excluded. An attenuation range of between −30 and −190 HU was then set [27,28]. Lastly, the images were checked and revised by operators to correct potential mistakes, and the total EAT volume was provided [12,29].

### 2.3. Statistical Analysis

For statistical analysis purposes, categorical data are expressed as a percentage and continuous data as the mean ± standard deviation. The χ^2^ test and two-sample *t* test were used to evaluate the differences in the categorical and continuous variables, respectively. The ln (CAC score +1) transformation was used to adjust for the rightward skew of the data and to reduce heteroscedasticity. Statistical significance was considered in the case of a *p* value < 0.05 (two-sided). In order to identify the variables associated with an increasing EAT volume and CAC, univariable and multivariable linear regression analyses were performed. Variables showing a *p* value < 0.05 in a univariable analysis were used to provide a multivariable model. All the analyses were performed using STATA version 18.

## 3. Results

The study population comprised 109 subjects with ages ranging from 18 to 74 years. Table 1 illustrates the main clinical indications for the PET/CT imaging test.

The baseline demographic and clinical characteristics of the study population according to CAC are illustrated in Table 2. In 38 (35%) patients, CAC was not detectable, and in 71 (65%), the CAC score was ≥1 with a mean ln (CAC +1) of 2.7 ± 2.6. The mean CAC score in patients with a CAC score ≥ 1 was 358 ± 743. Patients with a CAC score ≥ 1 were older than those without CAC (*p* < 0.001) and the prevalence of hypertension was higher in patients with detectable CAC compared to those without (*p* < 0.005). Similarly, patients with relevant coronary calcification (CAC score ≥ 160) were older than those with non-relevant coronary calcification (68 ± 9 vs. 55 ± 16 years, *p* < 0.005), while the other clinical characteristics did not differ between the two groups.

In the overall population, the mean EAT value was 99 ± 49 cm^3^, and it was higher in patients with CAC than in those without (110 ± 48 cm^3^ vs. 78 ± 43, *p* < 0.005). Within the limits of free breathing imaging, no focal tracer uptake on the EAT volume was observed.

Forty-five patients (41%) were treated with chemotherapy and/or radiotherapy before imaging. Of note, the ln (CAC +1) (2.8 ± 2.8 vs. 2.6 ± 2.5, *p* = 0.4) and EAT volume (102 ± 51 cm^3^ vs. 97 ± 47 cm^3^, *p* = 0.6) were not different between patients who underwent prior chemotherapy and/or radiotherapy and those who did not.

**Figure 1:**
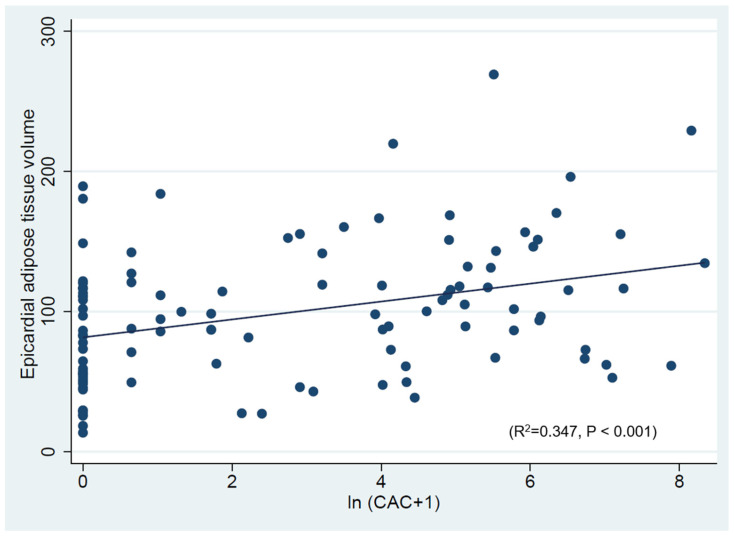
Correlation between epicardial adipose tissue (EAT) volume and coronary artery calcium (CAC) score.

The findings of the univariable and multivariable linear regression analyses are depicted in Table 3. Age, body mass index (BMI), hypertension, and ln (CAC +1) were significantly associated with increasing EAT values in the univariable analysis. In the overall population, a significant relationship (R^2^ = 0.347, *p* < 0.001) between the ln (CAC +1) and EAT volume was observed (Figure 1). However, when including clinical variables and CAC in the multivariable model, only age and BMI were independently associated with increasing EAT (R^2^ = 0.571, *p* < 0.001).

In the univariable analysis, age (*p* < 0.001) and hypertension (*p* < 0.005) were associated with an increasing CAC score. In the multivariable model, only age was independently associated with increasing CAC (*p* < 0.001).

**Figure 2:**
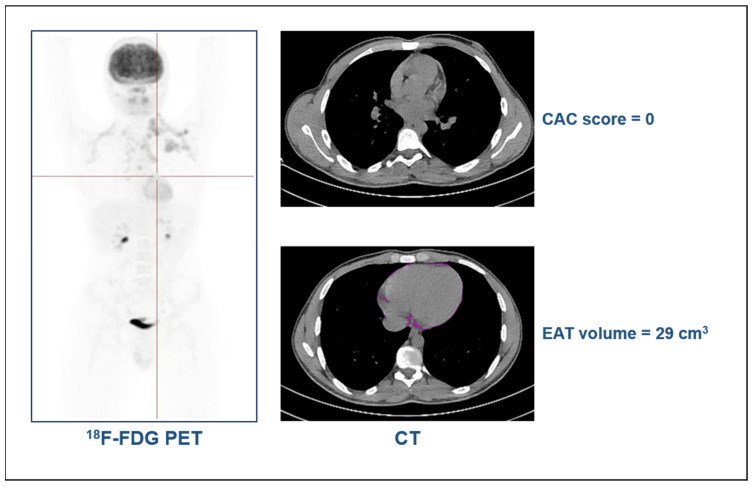
Case example of a 20-year-old man with Hodgkin’s lymphoma.

**Figure 3:**
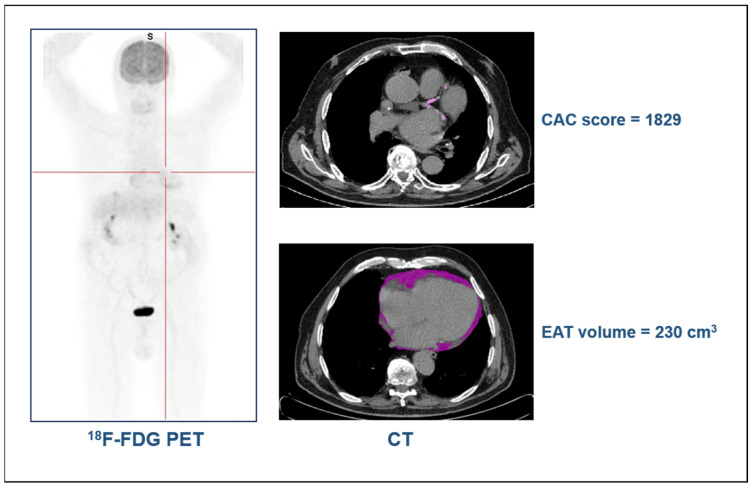
Case example of an 80-year-old man with colorectal cancer.

The examples of a 20-year-old man with a diagnosis of Hodgkin’s lymphoma, with a BMI of 21.8 and without risk factors, and an 80-year-old man with a diagnosis of colorectal cancer, with a BMI of 30 and hypertension, are presented in Figure 2 and Figure 3, respectively. As shown, in the old obese patient with hypertension, high values in the CAC score and EAT volume were measured, while in the young patient without risk factors, CAC was not detectable and the EAT volume values were negligible.

## 4. Discussion

The present study demonstrates for the first time the feasibility of the cost-effective evaluation of established markers of CAD, such as CAC scores and EAT, in patients without overt CAD undergoing whole-body PET–CT imaging for oncological reasons while also exploring the association of EAT volume with traditional cardiac risk factors. From an overall population of 109 patients, the majority (65%) demonstrated detectable coronary calcium. The role of CAC score measurements over cardiovascular risk factors has been established [30,31,32]. As expected, patients with detectable calcium burden were older than those without calcium. Starting from 50 years, age became the main cardiovascular risk factor due to the progressive accretion of atherosclerotic plaques over time [32,33]. The role of ageing in CAD development has been so widely demonstrated that novel concepts related to coronary calcium accumulation have been considered. In particular, coronary vascular age may be used as a surrogate for atherosclerotic burden [34]. Also, the higher prevalence of hypertension in patients with detectable CAC has been demonstrated [35,36,37]. Hence, the incorporation of CAC measurements into all non-contrast chest examinations may contribute to a significant jump forward in the early detection and treatment of CAD [29]. In our study population, age, BMI, hypertension, and CAC were associated with increasing EAT values. A significant but moderate relationship between EAT volume and CAC has been observed, with a large scattering. It is likely that a significantly larger patient cohort would have resulted in higher correlation. However, when the clinical variables and CAC were tested in a multivariable model, only age and BMI were independently associated with increasing EAT, suggesting that if on one hand there is a strong interplay between calcium burden and cardiac fat depot, on the other hand fat accumulation may have a direct link with other cardiovascular risk factors regardless of calcium load development. Therefore, even in cancer patients, the measurement of EAT volume does not seem redundant with respect to the evaluation of the CAC score for the purpose of estimating cardiovascular risk. Accordingly, our findings seem more relevant considering that EAT may contribute to the development of coronary vascular dysfunction before CAC accumulation [12]. The interplay between EAT and micro- and macro-vessel dysfunction has been demonstrated [38,39]. A recent cross-sectional study demonstrated that EAT volume was independently associated with CAC in a population of 409 patients with diabetes [40]. These latter findings are consistent with a previous investigation conducted on 127 patients that showed a significant relationship between EAT volume and diabetes, BMI, waist circumference, cholesterol, HDL-cholesterol, triglyceride levels, and the presence of metabolic syndrome [41]. On the other hand, EAT is at present attracting interest on the research ground as an early biomarker of atherosclerosis [11,40]. With regard to cancer populations, increasing EAT has recently been observed in breast cancer patients undergoing neoadjuvant chemotherapy [42]. The potential to expand cardiovascular assessment through whole-body unenhanced imaging using CT by evaluating both EAT volume and CAC scores can lead to a significant enhancement in clinical practice. This assumption should be read in light of the change in the natural history of oncological pathologies [43,44,45]. According to United States National Institute of Health [46], the amount of cancer survivors is expected to upsurge by 24.4%, to 22.5 million, by 2032. This trend, coupled with evidence indicating that cancer patients across all sites face an increased risk of cardiovascular death compared to the general population [3,47,48,49,50,51,52,53], underscores the imperative for establishing more streamlined cardiovascular care for cancer patients. This involves not only enhancing multidisciplinary collaboration among specialists including oncologists, cardiologists, and primary care physicians but also integrating diverse categories of information obtained from comprehensive imaging examinations.

The potential utility of CAC and EAT evaluation should be taken into consideration to stratify oncological patients referred to chemotherapy and/or radiotherapy at risk not only of adverse cardiovascular events for atherosclerotic development but also for cardiotoxicity [54,55]. This is even more relevant in the case of both occurrences. Furthermore, the potential to integrate CT with PET findings offers the possibility to look at metabolic data also for cardiovascular evaluation. Indeed, it has been demonstrated that an increased splenic ^18^F-FDG uptake is associated with cardiovascular events and the proinflammatory remodeling of circulating leukocytes, suggesting the presence of a cardio-splenic axis [56].

Although different studies have proposed EAT and CAC as markers of cardiac toxicity related to oncological therapy, in our population, these parameters were not different between patients who underwent prior chemotherapy and/or radiotherapy and those who did not. However, to deeply investigate the role of chemotherapy in the onset of cardiovascular disease, EAT and CAC scores should both be tested using serial imaging in a more homogeneous population.

Certain limitations of the present study should be acknowledged. Firstly, the retrospective nature of this single-center investigation contributes to the heterogeneous enrolled population, compounded by a small sample size. Therefore, a study in a homogeneous category of patients would have strengthened the value of the investigation. It should also be considered that 41% of our patients already received chemotherapy and/or radiotherapy before the imaging test. In spite of this, when patients were tested regarding the differences in CAC and EAT volumes according to previous chemotherapy, no statistical differences were found between the groups. Patients were tested under fasting conditions that lead to fatty acid metabolism activation, and ketosis was not measured. However, to our knowledge, there are no studies testing potential EAT characteristics changes in CT images under a fasting state. In addition, the CT imaging was performed without electrocardiographic gating or triggering, and cardiac motion artifacts might have affected the evaluation of the pericardium. The occurrence of cardiovascular events has not been considered in the analysis. The inclusion of patients without documented cardiovascular diseases and the absence of prognostic data and cardiac imaging results may also impact patient management. Furthermore, a control group of patients without cancer would have provided additional data and empowered the value of the reported findings. There is clear evidence on the prognostic role of EAT and CAC in patients with cardiovascular diseases [57,58]. However, further studies linking EAT volume and CAC to cardiovascular outcomes in cancer patients are needed. Finally, other risk factors should be considered as possible explanations for increased BMIs and EAT and consequently CAC in older oncological patients. In particular, sedentary lifestyles and a lack of physical activity, which are associated with unhealthy habits and hypercaloric diets, are additional influencing factors, leading to an increase in several chronic diseases such as high blood pressure, diabetes, obesity, and cholesterol, among others.

There is still an open debate regarding the choice of the best parameter that may indicate pathological adiposity [59]. Further studies are warranted to correlate the prevalence of sedentary behavior with pathological obesity parameters including not only BMI but also waist-to-hip ratio, EAT, and CAC in oncological patients with long life expectancies. Moreover, even if age and BMI are easier to measure than EAT, patients’ assessments may benefit from available data to provide a more holistic approach that looks at cancer and cardiovascular disease at a single time.

## 5. Conclusions

This study shows the feasibility of assessing, in a cost-effective manner, CAC scores and EAT volumes in patients referred to whole-body ^18^F-FDG PET–CT imaging, regardless of the main clinical question. This approach may allow the evaluation, at the same time, of cancer disease and atherosclerotic burden in a single test already included in the diagnostic program of oncological patients with radiation dose optimization and without additional costs. In the present investigation, increased age, hypertension, BMIs, and CAC scores are associated with EAT in oncological patients without known CAD.

## Figures and Tables

**Table 1:** Main clinical indications for PET–CT imaging.

Tumor	Patients (*n* = 109)
Hematological, *n* (%)	43 (39)
Urogenital, *n* (%)	22 (21)
Lung, *n* (%)	13 (12)
Breast, *n* (%)	8 (7)
Gastrointestinal, *n* (%)	7 (6)
Thyroid, *n* (%)	6 (6)
Melanoma, *n* (%)	3 (3)
Others, *n* (%)	7 (6)

**Table 2:** Demographic data and clinical characteristics according to CAC.

	All Patients (*n* = 109)	Without CAC (*n* = 38)	With CAC (*n* = 71)	*p* Value
Age (years)	58 ± 5	45 ± 17	64 ± 10	<0.001
Male gender, *n* (%)	53 (49)	19 (50)	37 (52)	0.83
Diabetes, *n* (%)	10 (9)	2 (5)	8 (11)	0.30
Hypertension, *n* (%)	47 (43)	9 (23)	38 (51)	<0.005
Hypercholesterolemia, *n* (%)	18 (17)	4 (11)	14 (20)	0.22
Smoking, *n* (%)	34 (31)	9 (23)	25 (35)	0.22
Family history of CAD, *n* (%)	18 (17)	6 (16)	15 (21)	0.50
Body mass index (kg/m^2^)	26.0 ± 4.1	25.6 ± 4.5	25.6 ± 4.5	0.99

Values are expressed as mean value ± standard deviation, as number (percentage) of subjects; CAC, coronary artery calcium; CAD, coronary artery disease.

**Table 3:** Linear regression analysis for prediction of increasing EAT volume in overall population.

	Univariable Analysis			Multivariable Analysis		
	SE	ß Coefficient	*p* Value	SE	ß Coefficient	*p* Value
Age	0.27	0.47	<0.001	0.32	0.45	<0.001
Male gender	9.3	−0.12	0.21			
Body mass index	0.99	0.31	<0.001	0.9	0.06	<0.001
Diabetes	16.1	0.13	0.18			
Hypertension	9.1	0.28	0.003	8.7	0.30	0.61
Hypercholesterolemia	12.4	0.17	0.70			
Smoking	10	0.14	0.14			
Family history of CAD	11.8	0.11	0.24			
Ln(CAC +1)	1.67	0.35	<0.001	1.83	0.12	0.25

EAT, epicardial adipose tissue, CAD, coronary artery disease, CAC, coronary artery calcium.

## Data Availability

The data presented in this study are available on request from the corresponding author. The data are not publicly available due to privacy restrictions.
